# Supplementary material for: Heparin-based hydrogel scaffolding alters the transcriptomic profile and increases the chemoresistance of MDA-MB-231 triple-negative breast cancer cells
Source: Biomater Sci. 2020 Feb 13;8(10):2786–96. doi: 10.1039/c9bm01481k (PMC7497406; doi:10.1039/c9bm01481k)
Supplement: Supplementary file 2 [file BM-008-C9BM01481K-s002.zip › Supplementary File 4/HP-B hydrogel + EGF (s)vControl/Pathways/my_analysis.Gsea.1545201170987/HALLMARK_ADIPOGENESIS.html]

Details for gene set HALLMARK\_ADIPOGENESIS[GSEA]

|  || Dataset | expr.class.cls#HYDEGF\_versus\_CONTROL.class.cls#HYDEGF\_versus\_CONTROL\_repos |
| Phenotype | class.cls#HYDEGF\_versus\_CONTROL\_repos |
| Upregulated in class | CONTROL |
| GeneSet | HALLMARK\_ADIPOGENESIS |
| Enrichment Score (ES) | -0.43915072 |
| Normalized Enrichment Score (NES) | -2.0908206 |
| Nominal p-value | 0.0 |
| FDR q-value | 0.0 |
| FWER p-Value | 0.0 |
Table: GSEA Results Summary

  

Fig 1: Enrichment plot: HALLMARK\_ADIPOGENESIS      
 Profile of the Running ES Score & Positions of GeneSet Members on the Rank Ordered List

  

| PROBE | DESCRIPTION (from dataset) | GENE SYMBOL | GENE\_TITLE | RANK IN GENE LIST | RANK METRIC SCORE | RUNNING ES | CORE ENRICHMENT || 1 | LIPE | na |  |  | 526 | 2.095 | -0.0143 | No |
| 2 | SLC1A5 | na |  |  | 679 | 1.922 | -0.0100 | No |
| 3 | PHLDB1 | na |  |  | 747 | 1.870 | -0.0015 | No |
| 4 | CDKN2C | na |  |  | 781 | 1.845 | 0.0085 | No |
| 5 | MYLK | na |  |  | 828 | 1.802 | 0.0177 | No |
| 6 | SLC19A1 | na |  |  | 985 | 1.698 | 0.0203 | No |
| 7 | SCARB1 | na |  |  | 994 | 1.690 | 0.0307 | No |
| 8 | ESRRA | na |  |  | 1494 | 1.435 | 0.0136 | No |
| 9 | SULT1A1 | na |  |  | 1665 | 1.367 | 0.0134 | No |
| 10 | GPHN | na |  |  | 1712 | 1.355 | 0.0197 | No |
| 11 | SLC25A10 | na |  |  | 1736 | 1.346 | 0.0271 | No |
| 12 | ITGA7 | na |  |  | 2100 | 1.239 | 0.0159 | No |
| 13 | ITSN1 | na |  |  | 2244 | 1.198 | 0.0161 | No |
| 14 | PEMT | na |  |  | 2435 | 1.144 | 0.0134 | No |
| 15 | RREB1 | na |  |  | 2559 | 1.113 | 0.0140 | No |
| 16 | GADD45A | na |  |  | 2798 | 1.052 | 0.0083 | No |
| 17 | ABCB8 | na |  |  | 2812 | 1.050 | 0.0143 | No |
| 18 | ADCY6 | na |  |  | 3056 | 0.990 | 0.0079 | No |
| 19 | UCP2 | na |  |  | 3188 | 0.963 | 0.0071 | No |
| 20 | DGAT1 | na |  |  | 3215 | 0.956 | 0.0119 | No |
| 21 | UCK1 | na |  |  | 3772 | 0.850 | -0.0119 | No |
| 22 | ANGPTL4 | na |  |  | 4183 | 0.784 | -0.0285 | No |
| 23 | LTC4S | na |  |  | 4210 | 0.780 | -0.0248 | No |
| 24 | NKIRAS1 | na |  |  | 4662 | 0.711 | -0.0440 | No |
| 25 | FAH | na |  |  | 4997 | 0.653 | -0.0574 | No |
| 26 | CPT2 | na |  |  | 5037 | 0.647 | -0.0553 | No |
| 27 | AGPAT3 | na |  |  | 5096 | 0.639 | -0.0543 | No |
| 28 | POR | na |  |  | 5102 | 0.637 | -0.0505 | No |
| 29 | ELMOD3 | na |  |  | 5300 | 0.605 | -0.0569 | No |
| 30 | SORBS1 | na |  |  | 5377 | 0.593 | -0.0571 | No |
| 31 | STAT5A | na |  |  | 5401 | 0.590 | -0.0546 | No |
| 32 | SLC5A6 | na |  |  | 5488 | 0.577 | -0.0554 | No |
| 33 | PPP1R15B | na |  |  | 5952 | 0.510 | -0.0765 | No |
| 34 | FZD4 | na |  |  | 5956 | 0.510 | -0.0734 | No |
| 35 | NMT1 | na |  |  | 6101 | 0.490 | -0.0778 | No |
| 36 | PTCD3 | na |  |  | 6346 | 0.458 | -0.0877 | No |
| 37 | AK2 | na |  |  | 6374 | 0.455 | -0.0862 | No |
| 38 | CYC1 | na |  |  | 6701 | 0.411 | -0.1007 | No |
| 39 | SAMM50 | na |  |  | 6908 | 0.384 | -0.1091 | No |
| 40 | ME1 | na |  |  | 7136 | 0.354 | -0.1188 | No |
| 41 | PREB | na |  |  | 7385 | 0.319 | -0.1298 | No |
| 42 | ACADS | na |  |  | 7614 | 0.288 | -0.1399 | No |
| 43 | ESYT1 | na |  |  | 7919 | 0.253 | -0.1543 | No |
| 44 | COQ3 | na |  |  | 7955 | 0.248 | -0.1545 | No |
| 45 | SPARCL1 | na |  |  | 8142 | 0.230 | -0.1628 | No |
| 46 | PIM3 | na |  |  | 8184 | 0.227 | -0.1636 | No |
| 47 | OMD | na |  |  | 8259 | 0.215 | -0.1661 | No |
| 48 | MGLL | na |  |  | 8440 | 0.196 | -0.1743 | No |
| 49 | ARAF | na |  |  | 8860 | 0.140 | -0.1954 | No |
| 50 | GPAM | na |  |  | 9094 | 0.116 | -0.2069 | No |
| 51 | QDPR | na |  |  | 9298 | 0.089 | -0.2170 | No |
| 52 | COL15A1 | na |  |  | 9638 | 0.056 | -0.2345 | No |
| 53 | COL4A1 | na |  |  | 9646 | 0.056 | -0.2345 | No |
| 54 | DHRS7B | na |  |  | 9667 | 0.052 | -0.2352 | No |
| 55 | IDH3G | na |  |  | 9687 | 0.051 | -0.2359 | No |
| 56 | BCL2L13 | na |  |  | 9827 | 0.031 | -0.2430 | No |
| 57 | CD302 | na |  |  | 10025 | 0.017 | -0.2532 | No |
| 58 | DHRS7 | na |  |  | 10107 | 0.004 | -0.2575 | No |
| 59 | PFKL | na |  |  | 10128 | 0.001 | -0.2585 | No |
| 60 | AIFM1 | na |  |  | 10142 | -0.001 | -0.2592 | No |
| 61 | ETFB | na |  |  | 10598 | -0.050 | -0.2828 | No |
| 62 | CHUK | na |  |  | 10609 | -0.052 | -0.2830 | No |
| 63 | CRAT | na |  |  | 10668 | -0.062 | -0.2856 | No |
| 64 | BAZ2A | na |  |  | 10963 | -0.101 | -0.3004 | No |
| 65 | VEGFB | na |  |  | 10970 | -0.102 | -0.3001 | No |
| 66 | HIBCH | na |  |  | 11001 | -0.107 | -0.3010 | No |
| 67 | ATL2 | na |  |  | 11065 | -0.116 | -0.3036 | No |
| 68 | STOM | na |  |  | 11213 | -0.124 | -0.3105 | No |
| 69 | TKT | na |  |  | 11286 | -0.135 | -0.3134 | No |
| 70 | ACADL | na |  |  | 11372 | -0.143 | -0.3170 | No |
| 71 | CD151 | na |  |  | 11390 | -0.145 | -0.3169 | No |
| 72 | LPL | na |  |  | 11671 | -0.184 | -0.3305 | No |
| 73 | HADH | na |  |  | 11730 | -0.192 | -0.3323 | No |
| 74 | SSPN | na |  |  | 12019 | -0.219 | -0.3460 | No |
| 75 | GPD2 | na |  |  | 12156 | -0.238 | -0.3517 | No |
| 76 | ACLY | na |  |  | 12160 | -0.238 | -0.3503 | No |
| 77 | APOE | na |  |  | 12183 | -0.241 | -0.3499 | No |
| 78 | COX8A | na |  |  | 12211 | -0.246 | -0.3498 | No |
| 79 | PQLC3 | na |  |  | 12277 | -0.257 | -0.3515 | No |
| 80 | GRPEL1 | na |  |  | 12492 | -0.285 | -0.3610 | No |
| 81 | RETSAT | na |  |  | 12706 | -0.315 | -0.3702 | No |
| 82 | NDUFS3 | na |  |  | 12718 | -0.316 | -0.3687 | No |
| 83 | IMMT | na |  |  | 12982 | -0.338 | -0.3804 | No |
| 84 | ACO2 | na |  |  | 13111 | -0.355 | -0.3848 | No |
| 85 | ARL4A | na |  |  | 13134 | -0.358 | -0.3837 | No |
| 86 | IDH3A | na |  |  | 13195 | -0.370 | -0.3845 | No |
| 87 | CS | na |  |  | 13254 | -0.378 | -0.3851 | No |
| 88 | PEX14 | na |  |  | 13302 | -0.386 | -0.3851 | No |
| 89 | DDT | na |  |  | 13433 | -0.408 | -0.3894 | No |
| 90 | DBT | na |  |  | 13526 | -0.418 | -0.3915 | No |
| 91 | SLC27A1 | na |  |  | 13553 | -0.421 | -0.3902 | No |
| 92 | UQCRC1 | na |  |  | 13620 | -0.428 | -0.3909 | No |
| 93 | CHCHD10 | na |  |  | 13791 | -0.458 | -0.3969 | No |
| 94 | RAB34 | na |  |  | 14215 | -0.506 | -0.4159 | No |
| 95 | ECH1 | na |  |  | 14216 | -0.507 | -0.4127 | No |
| 96 | ATP1B3 | na |  |  | 14257 | -0.514 | -0.4115 | No |
| 97 | ECHS1 | na |  |  | 14355 | -0.530 | -0.4132 | No |
| 98 | TST | na |  |  | 14375 | -0.533 | -0.4108 | No |
| 99 | GPX4 | na |  |  | 14401 | -0.538 | -0.4087 | No |
| 100 | ENPP2 | na |  |  | 14515 | -0.559 | -0.4110 | No |
| 101 | MCCC1 | na |  |  | 14575 | -0.570 | -0.4105 | No |
| 102 | ACADM | na |  |  | 14777 | -0.601 | -0.4172 | No |
| 103 | SCP2 | na |  |  | 15058 | -0.644 | -0.4278 | No |
| 104 | PFKFB3 | na |  |  | 15066 | -0.646 | -0.4241 | No |
| 105 | LPCAT3 | na |  |  | 15133 | -0.659 | -0.4233 | No |
| 106 | RTN3 | na |  |  | 15360 | -0.699 | -0.4307 | No |
| 107 | ADIPOR2 | na |  |  | 15521 | -0.725 | -0.4345 | Yes |
| 108 | COQ9 | na |  |  | 15525 | -0.726 | -0.4300 | Yes |
| 109 | SNCG | na |  |  | 15587 | -0.740 | -0.4285 | Yes |
| 110 | ACOX1 | na |  |  | 15588 | -0.740 | -0.4238 | Yes |
| 111 | SLC25A1 | na |  |  | 15598 | -0.742 | -0.4195 | Yes |
| 112 | UQCR11 | na |  |  | 15617 | -0.747 | -0.4157 | Yes |
| 113 | PPARG | na |  |  | 15629 | -0.748 | -0.4115 | Yes |
| 114 | MDH2 | na |  |  | 15652 | -0.754 | -0.4078 | Yes |
| 115 | MAP4K3 | na |  |  | 15779 | -0.780 | -0.4094 | Yes |
| 116 | REEP6 | na |  |  | 15838 | -0.790 | -0.4074 | Yes |
| 117 | CMBL | na |  |  | 15888 | -0.800 | -0.4049 | Yes |
| 118 | MTCH2 | na |  |  | 15889 | -0.800 | -0.3998 | Yes |
| 119 | TALDO1 | na |  |  | 16127 | -0.852 | -0.4068 | Yes |
| 120 | UBQLN1 | na |  |  | 16162 | -0.861 | -0.4031 | Yes |
| 121 | HSPB8 | na |  |  | 16267 | -0.891 | -0.4029 | Yes |
| 122 | DLD | na |  |  | 16317 | -0.903 | -0.3997 | Yes |
| 123 | MRPL15 | na |  |  | 16371 | -0.917 | -0.3966 | Yes |
| 124 | DHCR7 | na |  |  | 16459 | -0.938 | -0.3952 | Yes |
| 125 | ALDH2 | na |  |  | 16573 | -0.962 | -0.3949 | Yes |
| 126 | ALDOA | na |  |  | 16587 | -0.967 | -0.3895 | Yes |
| 127 | ELOVL6 | na |  |  | 16631 | -0.979 | -0.3855 | Yes |
| 128 | PPM1B | na |  |  | 16653 | -0.987 | -0.3802 | Yes |
| 129 | DECR1 | na |  |  | 16731 | -1.006 | -0.3779 | Yes |
| 130 | GPX3 | na |  |  | 16764 | -1.015 | -0.3731 | Yes |
| 131 | NDUFB7 | na |  |  | 16774 | -1.017 | -0.3670 | Yes |
| 132 | DRAM2 | na |  |  | 16840 | -1.040 | -0.3638 | Yes |
| 133 | BCL6 | na |  |  | 17079 | -1.109 | -0.3692 | Yes |
| 134 | C3 | na |  |  | 17200 | -1.144 | -0.3682 | Yes |
| 135 | SDHB | na |  |  | 17291 | -1.178 | -0.3654 | Yes |
| 136 | RNF11 | na |  |  | 17296 | -1.180 | -0.3581 | Yes |
| 137 | NDUFA5 | na |  |  | 17332 | -1.194 | -0.3523 | Yes |
| 138 | GHITM | na |  |  | 17403 | -1.215 | -0.3482 | Yes |
| 139 | DNAJB9 | na |  |  | 17463 | -1.242 | -0.3433 | Yes |
| 140 | PGM1 | na |  |  | 17488 | -1.254 | -0.3366 | Yes |
| 141 | PRDX3 | na |  |  | 17543 | -1.275 | -0.3313 | Yes |
| 142 | ATP5O | na |  |  | 17678 | -1.320 | -0.3299 | Yes |
| 143 | RIOK3 | na |  |  | 17725 | -1.347 | -0.3237 | Yes |
| 144 | YWHAG | na |  |  | 17793 | -1.380 | -0.3184 | Yes |
| 145 | ABCA1 | na |  |  | 17887 | -1.432 | -0.3141 | Yes |
| 146 | UQCRQ | na |  |  | 17923 | -1.453 | -0.3067 | Yes |
| 147 | DLAT | na |  |  | 17988 | -1.481 | -0.3006 | Yes |
| 148 | ACAA2 | na |  |  | 18083 | -1.534 | -0.2957 | Yes |
| 149 | UQCR10 | na |  |  | 18089 | -1.539 | -0.2861 | Yes |
| 150 | TANK | na |  |  | 18094 | -1.545 | -0.2765 | Yes |
| 151 | REEP5 | na |  |  | 18113 | -1.554 | -0.2675 | Yes |
| 152 | SDHC | na |  |  | 18153 | -1.583 | -0.2594 | Yes |
| 153 | SUCLG1 | na |  |  | 18207 | -1.604 | -0.2519 | Yes |
| 154 | JAGN1 | na |  |  | 18234 | -1.628 | -0.2429 | Yes |
| 155 | COQ5 | na |  |  | 18294 | -1.676 | -0.2353 | Yes |
| 156 | TOB1 | na |  |  | 18307 | -1.689 | -0.2251 | Yes |
| 157 | CCNG2 | na |  |  | 18336 | -1.703 | -0.2157 | Yes |
| 158 | G3BP2 | na |  |  | 18461 | -1.807 | -0.2106 | Yes |
| 159 | MGST3 | na |  |  | 18477 | -1.823 | -0.1998 | Yes |
| 160 | APLP2 | na |  |  | 18489 | -1.831 | -0.1886 | Yes |
| 161 | PDCD4 | na |  |  | 18548 | -1.865 | -0.1798 | Yes |
| 162 | NDUFAB1 | na |  |  | 18589 | -1.910 | -0.1697 | Yes |
| 163 | LAMA4 | na |  |  | 18606 | -1.932 | -0.1581 | Yes |
| 164 | CMPK1 | na |  |  | 18624 | -1.951 | -0.1466 | Yes |
| 165 | CAT | na |  |  | 18668 | -2.014 | -0.1359 | Yes |
| 166 | IDH1 | na |  |  | 18690 | -2.035 | -0.1240 | Yes |
| 167 | LIFR | na |  |  | 18713 | -2.068 | -0.1120 | Yes |
| 168 | DNAJC15 | na |  |  | 18747 | -2.108 | -0.1002 | Yes |
| 169 | PHYH | na |  |  | 18766 | -2.139 | -0.0875 | Yes |
| 170 | ANGPT1 | na |  |  | 18822 | -2.234 | -0.0761 | Yes |
| 171 | GBE1 | na |  |  | 18855 | -2.282 | -0.0632 | Yes |
| 172 | PLIN2 | na |  |  | 18894 | -2.342 | -0.0502 | Yes |
| 173 | SOD1 | na |  |  | 18910 | -2.370 | -0.0358 | Yes |
| 174 | IFNGR1 | na |  |  | 18912 | -2.376 | -0.0207 | Yes |
| 175 | COX7B | na |  |  | 19032 | -2.677 | -0.0098 | Yes |
| 176 | COX6A1 | na |  |  | 19097 | -2.897 | 0.0053 | Yes |
Table: GSEA details [plain text format]

  

Fig 2: HALLMARK\_ADIPOGENESIS      
 Blue-Pink O' Gram in the Space of the Analyzed GeneSet

  

Fig 3: HALLMARK\_ADIPOGENESIS: Random ES distribution      
 Gene set null distribution of ES for **HALLMARK\_ADIPOGENESIS**
